# PP2A Affects Angiogenesis via Its Interaction with a Novel Phosphorylation Site of TSP1

**DOI:** 10.3390/ijms25031844

**Published:** 2024-02-03

**Authors:** Zsófia Thalwieser, Márton Fonódi, Nikolett Király, Csilla Csortos, Anita Boratkó

**Affiliations:** Department of Medical Chemistry, Faculty of Medicine, University of Debrecen, Egyetem tér 1, H-4032 Debrecen, Hungary; thalwieser.zsofia@med.unideb.hu (Z.T.); fonodi.marton@med.unideb.hu (M.F.); csortos@gmail.com (C.C.)

**Keywords:** thrombospondin-1, protein phosphatase 2A, protein kinase C, angiogenesis, phosphorylation

## Abstract

Alterations in angiogenic properties play a pivotal role in the manifestation and onset of various pathologies, including vascular diseases and cancer. Thrombospondin-1 (TSP1) protein is one of the master regulators of angiogenesis. This study unveils a novel aspect of TSP1 regulation through reversible phosphorylation. The silencing of the B55α regulatory subunit of protein phosphatase 2A (PP2A) in endothelial cells led to a significant decrease in TSP1 expression. Direct interaction between TSP1 and PP2A-B55α was confirmed via various methods. Truncated TSP1 constructs were employed to identify the phosphorylation site and the responsible kinase, ultimately pinpointing PKC as the enzyme phosphorylating TSP1 on Ser93. The biological effects of B55α–TSP1 interaction were also analyzed. B55α silencing not only counteracted the increase in TSP1 expression during wound closure but also prolonged wound closure time. Although B55α silenced cells initiated tube-like structures earlier than control cells, their spheroid formation was disrupted, leading to disintegration. Cells transfected with phosphomimic TSP1 S93D exhibited smaller spheroids and reduced effectiveness in tube formation, revealing insights into the effects of TSP1 phosphorylation on angiogenic properties. In this paper, we introduce a new regulatory mechanism of angiogenesis by reversible phosphorylation on TSP1 S93 by PKC and PP2A B55α.

## 1. Introduction

Thrombospondins (TSPs) are calcium-binding matricellular glycoproteins expressed in various cell types. The non-structural matricellular proteins under physiological conditions are present at low concentrations in the extracellular matrix (ECM); however, their expression level can increase greatly due to tissue injuries or under certain pathological conditions [1]. The TSP family consists of five members that are classified into two subgroups according to their molecular structure and oligomeric properties. TSP1 and TSP2 are both homotrimeric proteins and share similar domain structures, while TSP3, TSP4, and TSP5 are all pentamers [2,3,4,5]. TSP1 and TSP2 protein structures include several thrombospondin type repeats (TSR); therefore, they belong to the TSR supergene family as well [6].

TSP1 was initially described as a secreted thrombin-sensitive protein component of activated platelets [7], but TSP1 is secreted by several cell types, including endothelial cells [8,9], fibroblasts [10,11], smooth muscle cells [10], pneumocytes [12], macrophages, monocytes [13], and certain tumor cells [14,15,16]. 

TSP1 can interact with a variety of proteins, receptors, or ligands; both extracellular and intracellular interactions of TSP1 have been reported recently [17,18,19]. Variable actions of TSP1 are strongly related to its binding partners in a given environment. Structural domains of TSP1 are responsible for the multiple and wide-spreading functions. Each TSP1 subunit consists of an amino-terminal (N-terminal) and a carboxyl-terminal (C-terminal) globular domain, a homology procollagen (PC) region, also known as von Willebrand C repeat (vWC), followed by three types of specific repeat sequences, namely type 1 (TSR1), type 2 (TSR2), and type 3 (TSR3). Binding sites for heparin and heparan sulfate proteoglycan, decorin, various integrins, proteases, cytokines, and growth factors are also present. The N-terminal domain was found to be important in cell attachment, spreading, proliferation, and migration via the interaction with integrins (α3β1, α4β1, α6β1, and α9β1), as well as in platelet aggregation via binding to fibrinogen and heparin [20,21,22]. Interaction partners of the TSR domains bring TSP1 into further cellular functions [23].

Different domains of TSP1 recognize several endothelial cell receptors, including a cluster of differentiation 36 (CD36) [24], a cluster of differentiation 47 (CD47), or integrin-associated protein (IAP) [25]; accordingly, these interactions influence the biological functions and behavior of endothelial cells (EC). 

TSP1 is best known as the first identified angiogenesis inhibitor protein [26], which inhibits EC migration and proliferation in a CD36-independent fashion [27]. Type 1 repeats are associated with fibroblast growth factor (FGF)-2- and vascular endothelial growth factor (VEGF)-induced angiogenesis inhibition. At the same time, inhibition of angiogenesis by TSP1 causes not only the inhibition of cell migration, but the inducement of apoptosis as well [3]. Furthermore, TSP1 plays an essential role in cell adhesion regulations, proliferation, migration, and cell–cell interactions [3,28]. The most studied interaction of the C-terminal region of TSP1 is with CD47. The binding of TSP1 to CD47 modulates nitric oxide (NO) signaling; therefore, vascular tone and biological processes like adhesion, migration, apoptosis, and proliferation are also affected.

Posttranslational modifications of TSP1 and their effect on TSP1-regulated processes are lesser known. The protein sequence of TSP1 bears several target sites for posttranslational modifications. Lys173, Lys262, Lys306, and Lys472 side chains were reported as potential ubiquitination sites [29,30]. The local protease activity and binding partners affect the concentration of TSP1, which is crucial in its function [31]. However, Ser/Thr phosphorylation of TSP1 has not been shown yet. 

Regulation of the phosphorylation level of different proteins is greatly clinically relevant as these modifications are crucial in all cell functions [32,33,34]. Protein phosphatase 2A (PP2A) is one of the major members of the serine/threonine-specific phosphatases (PSPs) [35]. PP2A is ubiquitously expressed in numerous cell types, and it is responsible for approximately 90% of the Ser/Thr dephosphorylation in cells [36,37,38]. PP2A activity plays an important role in several cellular functions, such as the control of cell metabolism [39], regulation of various biological processes, including transcription and translation, DNA replication, signal transduction, proliferation, migration, and apoptosis [40,41,42,43]. Furthermore, it has a tumor suppressor function as well [44]. A typical PP2A holoenzyme consists of a heterodimeric core (scaffolding “A” subunit and a catalytic “C” subunit) and a variable “B” regulatory subunit. “B” subunit families (B, B′, B″ and B‴) determine substrate specificity and localization of the holoenzyme. The B55α subunit containing PP2A holoenzyme has been extensively studied throughout the years. Our previous results have shown that this holoenzyme form of PP2A is an essential component in the EC barrier integrity, and the activity of the holoenzyme plays a crucial role in the functioning of adherent junctions [45,46,47]. Furthermore, PP2A activity affects angiogenesis in endothelial cells [48]. In the present work, reversible phosphorylation of TSP1 was studied in endothelial cells and it is shown that protein kinase C (PKC) and PP2A-B55α holoenzyme regulate the phosphorylation and dephosphorylation of TSP1 in endothelial cells.

## 2. Results

### 2.1. TSP1 Is Downregulated in B55α-Depleted Endothelial Cells

**Figure 1:**
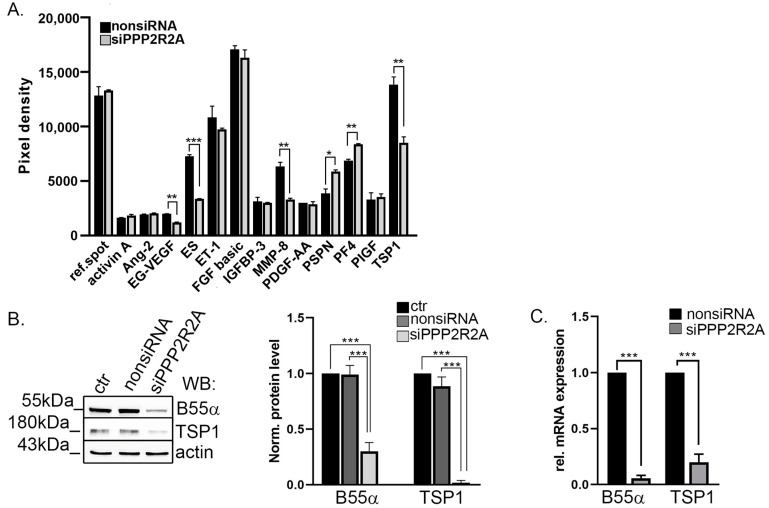
B55α depletion affects TSP1 in endothelial cells. (**A**) Cell lysates of non-targeting siRNA and B55α-specific siRNA (siPPP2R2A)-treated BPAEC were incubated on angiogenetic-specific antibodies containing proteome profiler array membrane. Quantification of the dots was performed using the ImageJ software (version 1.54h). Bars represent the mean of duplicated dots ± SD. Statistical analysis was performed by unpaired t-test (* *p* < 0.05, ** *p* < 0.01, and *** *p* < 0.001). (**B**) Control, non-targeting, and B55α-specific siRNA-treated BPAEC lysates were tested by Western blot. Quantitative analysis was performed by densitometry of the blots using actin bands for normalization. Statistical analysis was performed using ANOVA Tukey’s test (n = 3, *** *p* < 0.001). (**C**) qPCR measurements were made using mRNA from nonsiRNA and B55α-depleted (siPPP2R2A) BPAEC cells. Quantitative analysis of B55α and TSP1 signals are shown. GAPDH was used for mRNA-level normalization. Significant differences were determined by unpaired t-test (n = 3, *** *p* < 0.001).

To explore potential angiogenic pathways in which B55α is involved, we used siRNA-mediated transfection of bovine pulmonary artery endothelial cells (BPAEC). Optimal siRNA concentration was determined earlier, ensuring the effective modulation of target gene expression without compromising cell viability or inducing off-target effects [48]. After 72 h, lysates of nonsiRNA or B55α-specific siRNA (siPPP2R2A)-treated BPAEC were analyzed by Proteome Profiler Angiogenesis Array with 55 angiogenesis-related proteins (Supplementary Figure S1A). The analysis showed that 13 proteins were highly expressed in untreated BPAEC. B55α depletion significantly changed the protein level of endocrine gland-derived vascular endothelial growth factor (EG-VEGF), endostatin (ES), matrix metalloproteinase-8 (MMP-8), persephin (PSPN), platelet factor 4 (PF4), and TSP1. The TSP1 signal showed a large significant decrease in B55α-depleted cell lysate compared to the nonsiRNA transfected cell lysate (Figure 1A). The decreased level of TSP1 was confirmed by Western blot analysis of the control, non-, and B55α-specific siRNA-treated cells using a specific TSP1 antibody (Figure 1B). The protein level of B55α was reduced by 70% after silencing and, interestingly, the protein level of TSP1 decreased greatly as well. Total RNA was isolated from non-targeting and B55α siRNA-treated cells, and quantitative PCR measurements were performed. Depletion of B55α not only reduced the amount of TSP1 on the protein level, but on the mRNA level as well (Figure 1C). In addition to the pooled SMART siRNA, another specific siRNA was tested (PP2A-B55-α siRNA, sc-39185, Santa Cruz). We obtained similar results; however, the silencing efficiency was lower (Supplementary Figure S1B). Therefore, further experiments were performed using the pooled SMART siRNA. 

### 2.2. TSP1 Interacts with B55α Containing PP2A Holoenzyme in Endothelial Cells

**Figure 2:**
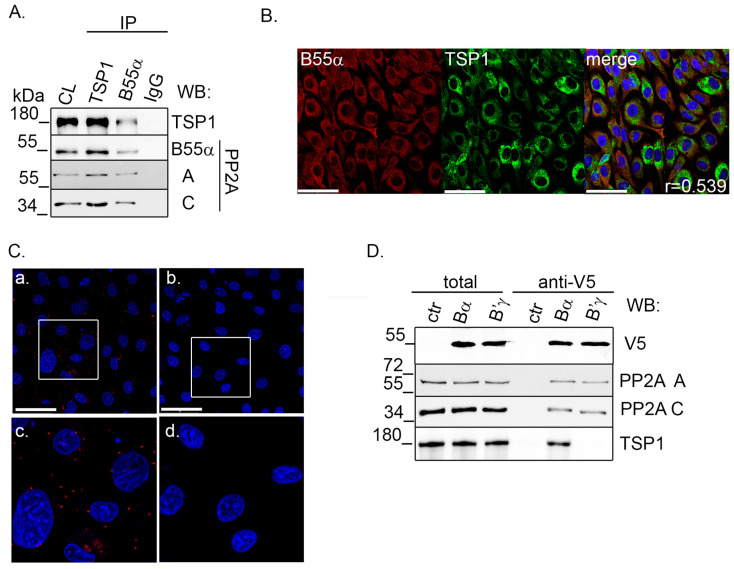
TSP1 interacts with PP2A-B55α holoenzyme in EC. (**A**) TSP1 or B55α was immunoprecipitated from BPAEC cells. Total cell lysate and immunocomplexes were tested for TSP1, PP2A A, B55α, and PP2A C subunits by Western blot. Rabbit IgG was applied as a control. (**B**) BPAEC cells were labeled with anti-TSP1 (green) and anti-B55α (red) antibodies, and immunofluorescent staining was performed. The nuclei of cells were labeled with DAPI (blue). Scale bars: 50 µm. (**C**) Duolink in situ PLA assay was made using anti-B55α and anti-TSP1 primary antibodies (**a**). As a negative control (**b**), cells were stained only with secondary antibodies. Nuclei were stained with DAPI (blue). Scale bars: 50 μm. The white rectangle area in panel (**a**) and panel (**b**) is shown as an enlarged panel in (**c**) and (**d**), respectively. (**D**) Non-transfected (ctr), pcDNA3.1 V5-His PP2A Bα (Bα) and pcDNA3.1 V5-His PP2A B’γ (B’γ) transfected cell lysates were incubated with anti-V5 agarose affinity gel. Total cell lysates and samples eluted from the affinity gel (anti-V5) were tested by Western blot using V5-tag, TSP1, PP2A A, and PP2A C subunit-specific antibodies.

Next, we examined whether TSP1 interacts with the B55α subunit containing the PP2A holoenzyme in EC. Immunoprecipitation (IP) was made using B55α- and TSP1-specific antibodies. Rabbit IgG was applied as a control. IP complexes were probed for antibodies raised against TSP1 and the subunits of the PP2A holoenzyme (Figure 2A). We found that TSP1 and B55α mutually co-immunoprecipitated with each other; moreover, we were able to detect the A and C subunits of PP2A. Immunofluorescent staining was carried out to examine the possible colocalization of B55α and TSP1. BPAEC cells were cultured on glass coverslips, and IF staining was carried out using specific B55α and TSP1 antibodies. Confocal images were taken and evaluated using the JACoP plugin in ImageJ software. Pearson’s coefficient demonstrates moderate colocalization (r = 0.4–0.59) of B55α and TSP1, and the merged image shows that they mainly colocalize in the cytoplasm of the cells (Figure 2B). 

To further verify the endogenous interaction of the proteins, a proximity ligation assay (PLA) was carried out. PLA detects protein-protein closeness within 40 nanometers with high sensitivity and specificity. The strong PLA signals obtained indicate the proximity of TSP1 and B55α in EC (Figure 2C). In negative control samples, which omitted primary antibodies, no signal was detected. The PP2A B subunit specificity of the newly described interaction was further examined by anti-V5 agarose affinity gel. Endothelial cells were transfected with pcDNA3.1-V5-His PP2A B55α or pcDNA3.1-V5-His PP2A B’γ constructs to produce V5-tagged B55α and B’γ proteins. Then, V5-tagged recombinant proteins (V5-B55α and V5-B’γ) were purified on an anti-V5 affinity gel. Total samples as well as affinity purified samples were tested by Western blot (Figure 2D). The results confirmed the successful overexpression and purification of V5-tagged B55α and B’γ subunits. As expected, both subunits interacted with PP2A A and C subunits, but TSP1 interacted only with B55α as no interaction was detected with B’γ. These results show that TSP1 specifically interacts with the B55α subunit containing PP2A holoenzyme.

### 2.3. Modulation of PKC Activity Regulates TSP1

**Figure 3:**
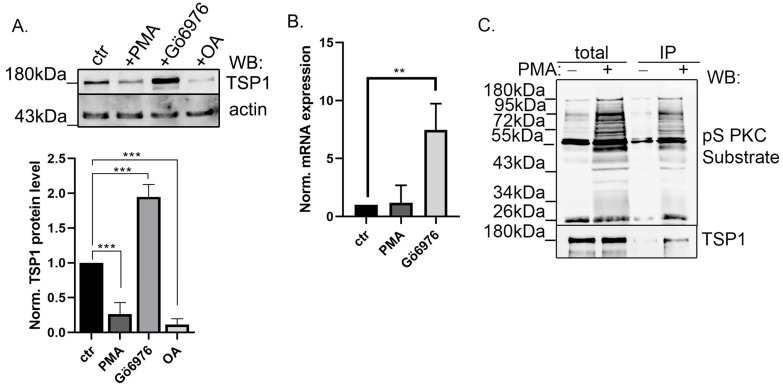
TSP1 is a PKC substrate (**A**) BPAEC cells were treated with 50 nM PMA, 1 μM Gö6976, or 5 nM okadaic acid for 12 h. Cell lysates were tested by Western blot using TSP1- and actin-specific antibodies. Quantitative analysis of TSP1 signals is also shown. Actin signals were used for protein level normalization. Statistical analysis was conducted by the ANOVA Dunnett test (n = 3, *** *p* < 0.001). (**B**) qPCR analyses were performed using isolated mRNA from control, PMA, or Gö6976-treated cells. GAPDH signals were used for mRNA-level normalization. Statistical analysis was conducted using the ANOVA Dunnett test (n = 3, ** *p* < 0.01). (**C**) Immunoprecipitation was made from control and PMA-treated endothelial cells using a phospho-Serine PKC substrate-specific antibody. Total lysates and the immunocomplexes were tested with phospho-Serine PKC substrate and TSP—specific antibodies.

The specific interaction of TSP1 with PP2A, a protein phosphatase, suggests reversible phosphorylation and interaction of TSP1 with a protein kinase as well. Therefore, first, the long-term effect of protein kinase A (PKA) and PKC activation and inhibition was tested on the TSP1 protein level (Supplementary Figure S2). Forskolin (PKA activator) and H89 (PKA inhibitor) had no effect on TSP1 protein expression; however, the activation or inhibition of PKC remarkably changed the TSP1 level. Therefore, we focused on further studying the possible regulatory role of PP2A and PKC on TSP1. Endothelial cells were treated with the PKC activator phorbol myristate acetate (PMA), Gö6976 (a PKC inhibitor), and okadaic acid (OA), which is a PP2A inhibitor. Samples were tested with TSP1- and actin-specific antibodies by Western blot (Figure 3A). Modulation of PKC activity strongly influenced the protein level of TSP1. While activation by PMA challenge decreased the TSP1 protein level in EC, inhibition by Gö6976 treatment significantly increased it. On the other hand, inhibition of PP2A by OA treatment, similar to B55α depletion, decreased the protein level of TSP1. TSP1 expression of the treated cells at the mRNA level was also analyzed by qPCR. TSP1 mRNA level was enhanced due to the Gö6976 treatment, but no significant change was detected in the PMA-treated cells compared to the control sample (Figure 3B). This suggested that reversible phosphorylation of TSP1 may play an important role in its regulation. PKC phosphorylation of TSP1 was further investigated by immunoprecipitation performed from control and PMA-treated BPAEC cells using phospho-Ser PKC substrate antibody that recognizes PKC phosphorylated substrates (Figure 3C). Total lysates and IP-complexes were tested with phospho-Ser PKC substrate- and TSP1-specific antibodies. We detected some TSP1 in the IP samples of control cells, implying a low phosphorylation level form of TSP1 present without PKC activation. A rather strong signal was found in PMA-treated samples, suggesting that TSP1 can be phosphorylated by PKC on a serine amino acid residue. 

### 2.4. PKC Phosphorylates TSP1 on Ser93 Residue

**Figure 4:**
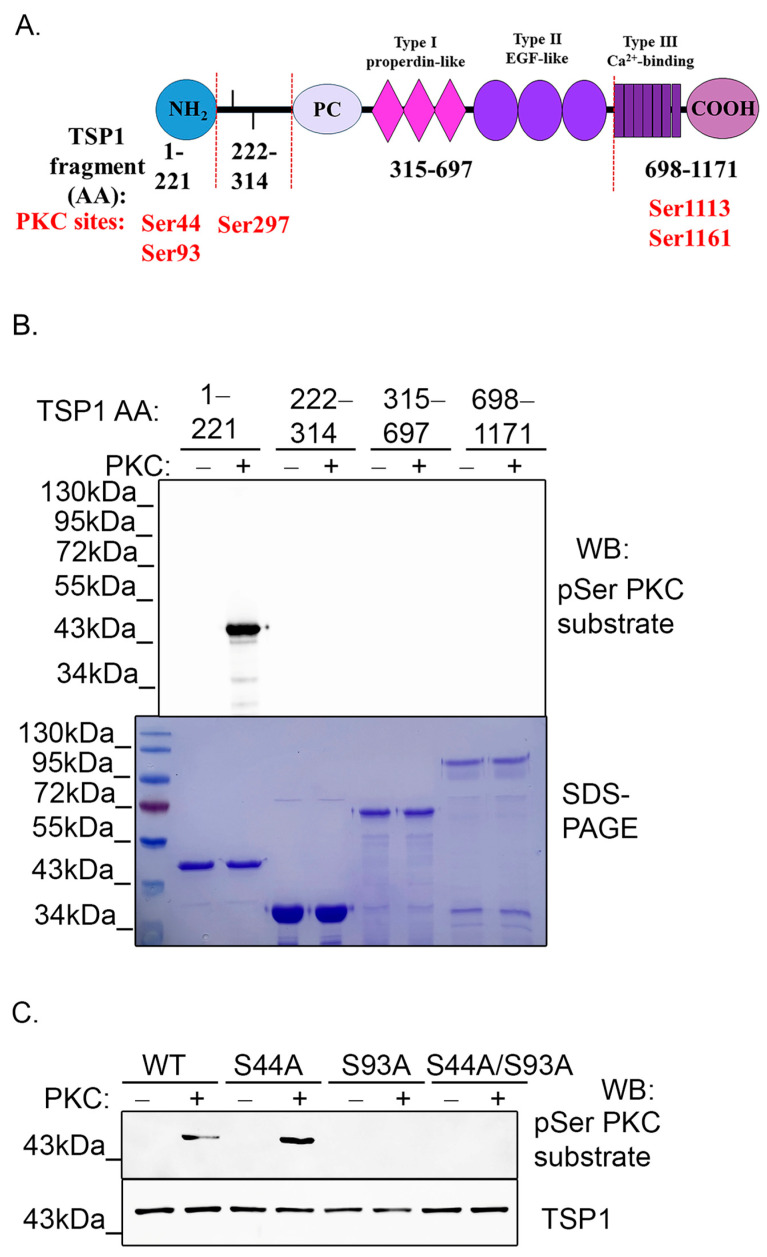
PKC phosphorylates TSP1 on Ser93 side chain (**A**) Schematic representation of the potential PKCα phosphorylation sites (red) in the TSP1 protein (GPS 5.0 software). Considering the TSP1 domain structure, shorter TSP1 fragments were created according to the indicated amino acids. NH_2_: globular N-terminal domain, PC: procollagen, thrombospondin repeats: type I, type II, and type III, and COOH: C-terminal domain. (**B**) GST-tagged TSP1 fragment proteins were purified on glutathione Sepharose beads and incubated with or without active PKC for 2 h at 30 °C. Samples were tested by Western blot using a phospho-Serine PKC substrate-specific antibody and by Coomassie Blue staining of SDS-PAGE. (**C**) Recombinant GST-TSP1^1–221^ WT, GST-TSP1^1–221^ S44A, GST-TSP1^1–221^ S93A, and GST-TSP1^1–221^ S44A/S93A proteins were purified and incubated with or without active PKC. Phosphorylation of the proteins was tested with phospho-Ser PKC substrate antibody by Western blot.

Next, we intended to identify the side chain that can be phosphorylated by PKC. Group-based Prediction System (GPS) 5.0 phosphorylation prediction software [49] showed five high score potential phosphorylation sites of the full-length TSP1, namely Ser44, Ser93, Ser297, Ser1113, and Ser1163. Considering the domain structure of TSP1, several shorter fragments were cloned into a pGEX-4T-2 vector to produce glutathione S-transferase (GST)-tagged truncated TSP1 proteins (Figure 4A). Recombinant protein production and purification of GST-TSP1^1–221^, GST-TSP1^222–314^, GST-TSP1^315–697^, and GST-TSP1^698–1171^ fragments were optimized and then, in vitro PKC phosphorylation assays were performed using the purified GST-tagged fragment proteins. Fragments were incubated with or without active PKCα enzyme for 2 h. The phosphorylated samples were tested with a phospho-Ser PKC substrate-specific antibody by Western blot (Figure 4B). PKC exclusively phosphorylated the first fragment (1–211aa), suggesting that the potential phosphorylation site of TSP1 can be Ser44 and/or Ser93. A recombinant single and double phosphonull mutant of TSP1^1–221^ fragments were made in order to confirm these potential phosphorylation site(s) of the TSP1. Ser44 and/or Ser93 side chains were changed to alanine by site-directed mutagenesis and confirmed by sequencing. An in vitro PKC phosphorylation assay was carried out on GST-TSP1^1–221^ S44A, GST-TSP1^1–221^S93A, and GST-TSP1^1–221^S44A/S93A mutants. Wild type (WT) GST-TSP1^1–221^ was used as control. Samples were tested by Western blot using a phospho-Ser PKC substrate-specific antibody. GST-TSP1^1–221^ wild type and GST-TSP1^1–221^ S44A proteins were phosphorylated by PKC, while S93A mutation containing GST-TSP1^1–221^ and GST-TSP1^1–221^ S44A/S93A mutants showed no phosphorylation (Figure 4C). These results led us to the conclusion that Ser93 of TSP1 is phosphorylated by PKC.

**Figure 5:**
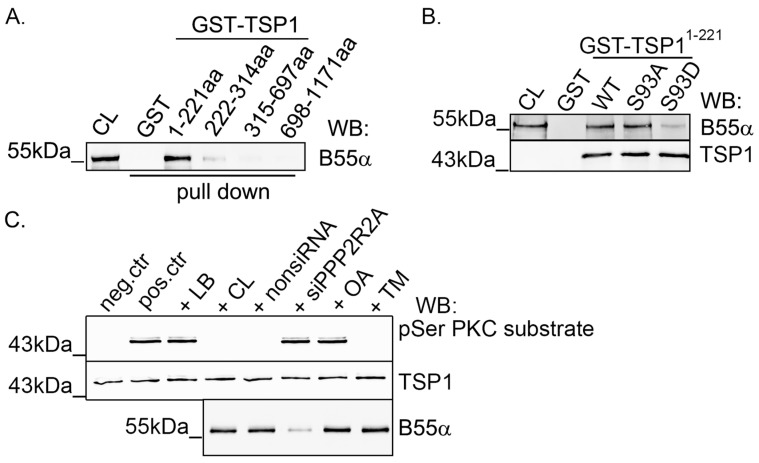
PP2A is involved in TSP1 dephosphorylation. (**A**) The pull down experiment from BPAEC cell lysate was carried out using bacterially expressed and purified recombinant GST and GST-TSP1 fragments. The eluted samples were tested for B55α in a Western blot experiment. (**B**) Immobilized GST-tagged TSP1^1–221^ wild type, S93A, and S93D mutant proteins were incubated with BPAEC cell lysate in a pull down experiment. Samples were tested using B55α-specific and TSP1 (loading control) antibodies by Western blot. (**C**) Dephosphorylation of TSP1 was carried out by an addition of a lysis buffer (LB) (negative control of dephosphorylation), BPAEC cell lysate (CL), nonsiRNA, siPPP2R2A transfected cell lysates, BPAEC lysates pretreated with 5 nm OA, or 1 μm tautomycetin (TM) to the in vitro PKC phosphorylated phospho-TSP1 (1–221) (pos. ctr). TSP1 (1–221) in the negative control of phosphorylation (neg. ctr) was not phosphorylated by in vitro PKC treatment. The efficiency of the silencing of B55α was verified by checking the B55α protein level of the samples. Dephosphorylation was analyzed by testing the phosphorylation level of TSP1 (1–221) using a phospho-Ser PKC substrate antibody and a TSP1 antibody (loading control).

In light of the above results, the interaction between B55α and TSP1 was examined with a pull down assay using the truncated fragments of TSP1. Immobilized fragment proteins were incubated with BPAEC lysate overnight and the pull down samples were analyzed by Western blot using a B55α-specific antibody. B55α was found to interact with the PKC phosphorylation site containing an N-terminal fragment (GST-TSP1^1–221^) (Figure 5A). 

To study the effect of the phosphorylation state of Ser93 on the interaction between TSP1 and B55α, pull down assays were performed with immobilized GST-TSP1^1–221^ WT, -S93A, and -S93D proteins. A Western blot analysis of the pull down samples showed that B55α interacted with all three TSP1 fragments, but the least amount of B55α was obtained with the phosphomimic GST-TSP1^1–221^ S93D compared to the wild type or phospho-null GST-TSP1^1–221^ mutant (Figure 5B). If phospho-TSP1 is a substrate of PP2A, one could expect the binding of the recombinant phosphomimic TSP1 fragment to B55α. However, although we detected some binding, fewer B55α proteins were bound to the immobilized GST-tagged phosphomimic fragment. This raises the following question: Can TSP1 be a substrate for PP2A or not? Next, we analyzed whether PP2A is involved in phospho-TSP1 dephosphorylation using a phosphorylated TSP1 fragment instead of the phosphomimic form of TSP1. Phosphatase assay was made using the in vitro PKC phosphorylated GST-TSP1^1–221^ fragment as a substrate. The phosphorylation level of the TSP1 fragment was analyzed by Western blot (Figure 5C). TSP1 was dephosphorylated with BPAEC cell lysate, nonsiRNA treated cell lysate, or cell lysate with the addition of a PP1 inhibitor, tautomycetin (TM; however, depletion of the B55α subunit or inhibition of PP2A enzyme activity using OA avoided the dephosphorylation. These results imply that the PP2A-B55α holoenzyme is responsible for the dephosphorylation of phospho-Ser93 TSP1. The dephosphorylation assays with the phosphorylated TSP1 substrate in the presence of specific phosphatase inhibitors show the enzyme-substrate interaction. The rather unexpected behavior of the phosphomimic mutant (Figure 5B) can be simply the consequence of the extra negative charge. It is possible that the negative charge of the truncated mutant results in a conformation change of the mutant that is different from the phosphorylated TSP1 substrate.

### 2.5. B55α Is Essential for TSP1 Upregulation in Wound Healing

**Figure 6:**
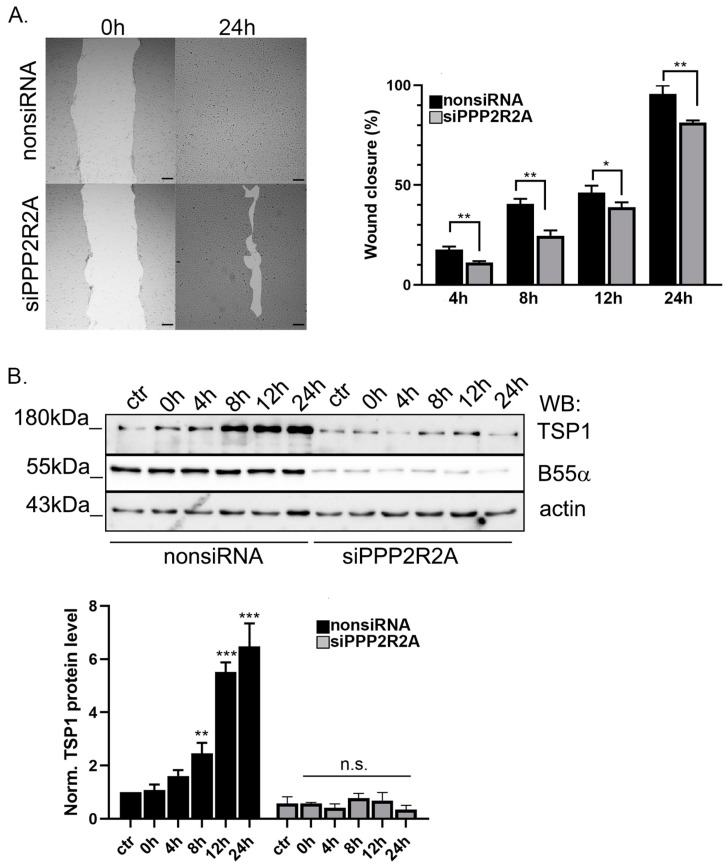
B55α depletion affects TSP1 protein level during wound healing. (**A**) NonsiRNA and B55α (siPPP2R2A)-depleted BPAEC cells were seeded in 12 well plates. Confluent monolayers of the cells were scratched with a 1 mL pipet tip. The wound healing of the cells was followed by imaging at indicated timepoints. The wound closure was evaluated by the ImageJ software. Statistical analysis was performed using a t-test (n = 3, * *p* < 0.05, ** *p* < 0.01). (**B**) Unscratched (ctr) and scratched nonsiRNA or B55α-depleted BPAEC cells (siPPP2R2A) were washed with 1× PBS and then collected in a protease inhibitor cocktail containing a lysis buffer at indicated timepoints. Samples were tested by Western blot using TSP1-, B55α-, and actin-specific antibodies. Actin bands were used for normalization in densitometric analysis. Statistical analysis was performed using an ANOVA Tukey’s test (n = 3, n.s: not significant, ** *p* < 0.01, *** *p* < 0.001).

To learn about the physiological consequences of B55α depletion, a scratch assay was made using non-targeting siRNA and B55α-specific siRNA-treated endothelial cells (Figure 6A). The results showed that the wound healing ability of the B55α-depleted cells was slower compared to the control cells. Protein levels were also studied, and scratched nonsiRNA and B55α-specific siRNA treated cells were collected at different timepoints and then tested by Western blot with TSP1-, B55α-, and actin-specific antibodies (Figure 6B). It is known from the literature, that TSP1 protein level increases during wound healing [50]. Indeed, the Western blot results confirmed that TSP1 level increased during wound closure in nonsiRNA-treated cells, but no TSP1 protein level change was found in cells lacking B55α. These results show that TSP1 is regulated during wound healing by PP2A.

### 2.6. B55α Is Involved in Spheroid Stability and Tube Formation of EC 

**Figure 7:**
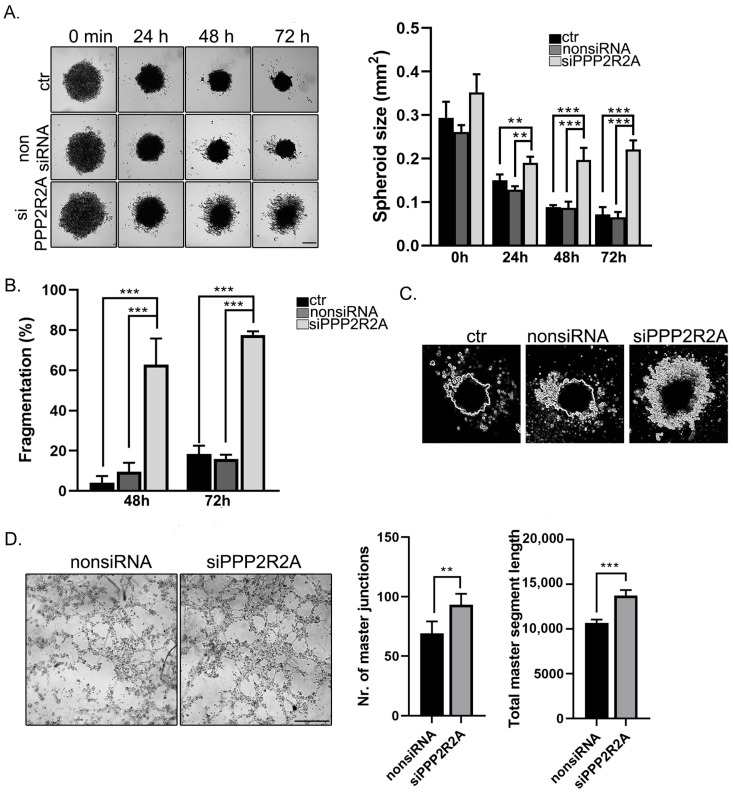
PP2A affects the spheroid and tube formation of EC. (**A**) Control, non-targeting, or B55α siRNA-treated BPAEC cells were incubated with NanoShuttle-PL magnetic beads overnight. The magnetized cells were seeded into a cell-repellent plate and then incubated on a magnetic plate for 24 h to initialize spheroid formation. After the spheroids were formed, the magnetic plate was removed (0 min), and images were taken at indicated timepoints. Sizes of spheroids were evaluated using ImageJ. Statistical analysis was performed using an ANOVA Tukey’s test (n = 3, ** *p* < 0.01, *** *p* < 0.001). (**B**) Fragmentation of spheroids (%) was calculated based on released cells or clusters/total area ×100 of the spheroid. (**C**) Representative images of released cells (white) and the core structure of spheroids are shown. (**D**) NonsiRNA and PPP2R2A siRNA transfected BPAEC cells were plated on a Matrigel-coated µ-slide angiogenesis plate, and tube formation was followed. Representative images were taken 3 h after seeding. Scale bar: 500 µm. The evaluation was made using the Angiogenesis Analyzer tool in ImageJ. Statistical analysis of tube formation was performed using an unpaired t-test (n = 3, ** *p* < 0.01, *** *p* < 0.001).

Angiogenic properties like spheroid or tube formation of cells were analyzed next. For the 3D cell culturing, a 96-well Bioprinting Kit (Greiner Bio-One, Kremsmünster, Austria) was used. The cells were magnetized by NanoShuttle-PL; therefore, they were able to mimic the native tissue environment and form 3D formation within hours in vitro. NanoShuttle magnetic beads were added to the control and silenced cells. The cells aggregated and formed a 3D spheroid form due to the magnetic forces. Images were taken at different timepoints to follow the changes of the spheroids after their removal from the magnetic field (zero time) (Figure 7A). The size of the spheroids was evaluated by the ImageJ software. The efficiency of silencing was confirmed by Western blot using the magnetized cells (Supplementary Figure S3A). Depletion of B55α had an extremely large effect on the spheroid formation of the endothelial cells. Although the overall size of the spheres formed from B55α-lacking cells was larger during the entire time period of the experiment, those spheroids started to disintegrate/fragmentate after 48 h. Fragmentation of spheroids was quantified at 48-h and 72-h timepoints (Figure 7B). In the absence of B55α, the disintegration of the spheroids was more dramatic compared to the spheroids formed from control or nonsiRNA-treated cells, which were rather stable. Tube formation ability was also studied using a Matrigel 3D matrix. Cells were seeded on a Matrigel µ-Slide angiogenetic plate and tube formation was followed over time. Figure 7C shows that the B55α-silenced cells already had tube-like structures 3 h after seeding. An evaluation of tube lengths and branching points was also performed in control and depleted cells. The results show that the B55α-lacking cells had significantly more branching points and total segment length than the control cells (Figure 7C). 

### 2.7. TSP1 S93D Mutants Inhibits In Vivo Angiogenesis

**Figure 8:**
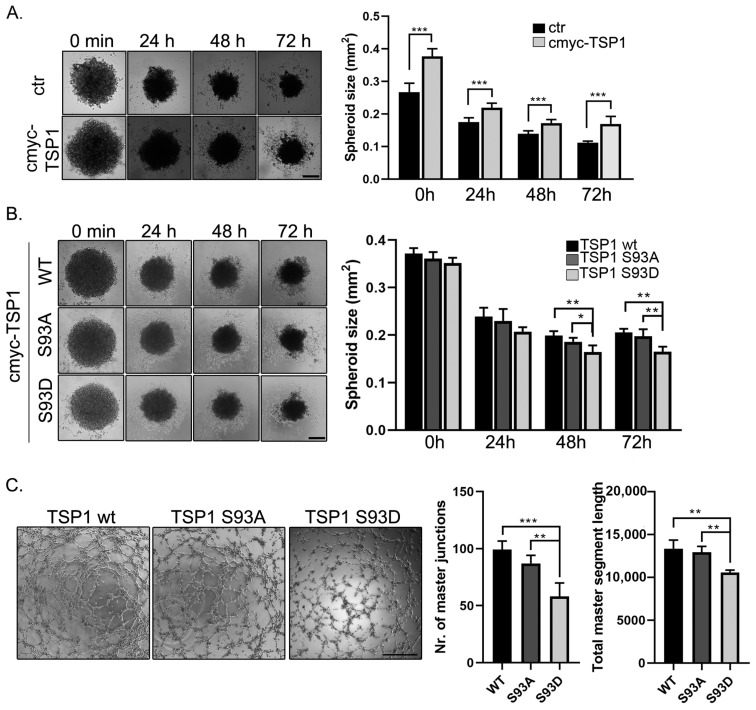
TSP1 S93D mutants affect spheroid and tube formation of EC. (**A**) Lipofectamine control (ctr) and pcDNA3.1 myc-HisA TSP1 transfected BPAEC cells were incubated with NanoShuttle-PL magnetic beads overnight. Spheroid formation was followed over time. Sizes of spheroids were evaluated using ImageJ. Statistical analysis was performed using an ANOVA Tukey’s test (n = 3, *** *p* < 0.001). (**B**) Spheroid size of pcDNA-TSP1 wt, S93A, or S93D transfected cells were analyzed. Images were taken at indicated timepoints. The evaluation was performed as mentioned earlier (n = 3, * *p* < 0.05, ** *p* < 0.01). (**C**) pcDNA-TSP1 wt, S93A, or S93D transfected BPAEC cells were plated on a Matrigel-coated μ-Slide angiogenesis plate. After the tube formation of the different TSP1 construct overexpressed cells, images were taken at indicated timepoints. Representative images were taken 5 h after seeding. The evaluation was made using the Angiogenesis Analyzer tool in ImageJ. Significant changes in tube formation ability were determined by an ANOVA Tukey’s test (n = 3, ** *p* < 0.01, *** *p* < 0.001). Scale bar: 500 µm.

Our experiment showed a strong connection between PP2A and TSP1, but it is important to note that the observed effects cannot be solely attributed to the downregulation of TSP1 caused by PP2A depletion as many other proteins are affected by PP2A. Therefore, the effect of TSP1 overexpression on spheroid formation was analyzed. Endothelial cells were transfected with pcDNA3.1myc-His-TSP1 plasmid, and spheroid formation was initiated 24 h after transfection as described before. Our results show that the TSP1 overexpressing cells formed significantly larger spheroids compared to controls at time 0, despite the same number of cells used for each sample (Figure 8A). This size difference was maintained for 72 h. The possible effect of the phosphorylation of S93 of the TSP1 protein, phosphomimic (S93D), and phosphonull (S93A) TSP1 full-length constructs were also made, and spheroid formation of wt and mutant overexpressing cells was studied (Supplementary Figure S3B). We found that TSP1-S93D overexpressing cells formed smaller and more compact spheroids at 48 and 72 h timepoints (Figure 8B). In addition, the tube formation ability of cells overexpressing the different TSP1 forms was studied as well (Figure 8C). An evaluation of the capillary network of the TSP1 wild type, S93A, and S93D overexpressing cells showed that phosphomimic TSP1 mutant transfected cells had significantly fewer total master junctions and shorter total segment lengths than wild type or S93A mutant expressing cells. These results suggest that PKC phosphorylation may affect how TSP1 modulates the angiogenic properties of EC.

## 3. Discussion

Angiogenesis is a tightly regulated process involving a balance between pro-angiogenic and anti-angiogenic signals. Ser/Thr phosphatases are involved in modulating signaling pathways that regulate endothelial cell behavior, migration, proliferation, and tube formation—key processes in angiogenesis. PP2A is known to dephosphorylate various substrates involved in angiogenic pathways, such as Akt [51] or endothelial nitric oxide synthase (eNOS) [52,53]; moreover, it is involved in the VEGF pathway as well [54]. Several research studies on PP2A focus on understanding its role as a tumor suppressor and its potential role as a therapeutic target in tumor biology, where the extent of angiogenesis is often used as a diagnostic and prognostic marker [55]. The development of a vascular network is crucial for supplying tumors with nutrients and oxygen, facilitating their growth and progression. 

Our study aimed to explore potential angiogenic pathways involving the PP2A-B55α holoenzyme. Angiogenesis-related proteins were analyzed using a Proteome Profiler Angiogenesis Array and notably, the protein level of TSP1 significantly decreased upon B55α depletion, confirmed by Western blot and mRNA analysis. TSP1 is one of the most potent regulators of angiogenesis. It plays diverse and crucial roles in various physiological and pathological processes (reviewed in [56]). TSP1 has been shown to have both pro- and anti-angiogenic effects, depending on the context. It can inhibit angiogenesis by directly interacting with endothelial cells and inhibiting their migration and proliferation [57,58,59]. On the other hand, it can also promote angiogenesis indirectly by modulating the activity of other growth factors and signaling pathways [60,61,62]. Understanding its antiangiogenic and proangiogenic properties provides valuable insights into the mechanisms controlling blood vessel development, which is essential for normal physiological processes such as embryonic development, wound healing, and tissue repair [63]. Some of the well-known posttranslational modifications of TSP1 include glycosylation and disulfide bond formation, which collectively contribute to the structural and functional diversity of TSP1 [23,64,65]. We found a direct interaction between TSP1 and the Ser/Thr phosphatase PP2A. Immunofluorescent staining and a proximity ligation assay supported the colocalization and proximity of the B55α subunit of PP2A and TSP1 in the cytoplasm of cells. Immunoprecipitation experiments demonstrated mutual binding between TSP1 and B55α, along with the detection of PP2A A and C subunits. We hypothesized that PP2A may target TSP1 for dephosphorylation, but no specific Ser/Thr phosphorylation site of TSP1 was known.

As protein phosphatases play a crucial role in counteracting the actions of protein kinases, we employed and tested challenges that affect protein kinases. We showed that PKC activation or inhibition significantly altered TSP1 levels, implicating PKC in TSP1 regulation. Immunoprecipitation experiments revealed that TSP1 is phosphorylated by PKC on a serine residue. Phosphorylation prediction software identified several potential phosphorylation sites, leading to in vitro assays that confirmed PKC phosphorylation of TSP1 at Ser93 in the N-terminal domain.

The specificity of PP2A for its substrates is determined by the B regulatory/targeting subunits associated with the catalytic subunit [66]. The B subunits of PP2A recognize specific amino acid sequences or motifs in substrate proteins, contributing to the precise dephosphorylation of target substrates. Pull-down assays identified the interaction between B55α and the PKC phosphorylation site containing the N-terminal fragment of TSP1. Moreover, our in vitro experiments suggest that PP2A is responsible for the dephosphorylation of phospho-Ser93 TSP1. The N-terminal domain of TSP1 contains binding sites for cell surface receptors, including CD36 [67]. Upon binding of CD36 to TSR type I repeat of TSP1, CD36 triggers signals with antiangiogenic effects, leading to the initiation of endothelial cell apoptosis [68,69]. CD36 is commonly found in human microvascular endothelial cells, but it is not present in large vessel endothelial cells like human umbilical vein endothelial cells (HUVEC) [70]. TSP1 has been shown to bind to integrins via its N-terminal domain, influencing cell adhesion and migration [20]. Integrin-mediated signaling is essential for processes like angiogenesis, wound healing, and tissue remodeling. The N-terminal domain is also susceptible to proteolytic cleavage by enzymes such as matrix metalloproteinases (MMPs) [71]. This cleavage can generate smaller fragments, and the resulting proteolytic fragments may have distinct functions from the full-length TSP1 molecule. 

The level of TSP1 can be influenced by various factors, and its expression is regulated by a complex interplay of signals in different physiological and pathological contexts. Hypoxia, or low oxygen levels, can upregulate TSP1 expression, and TSP1 has been implicated in the regulation of transforming growth factor-β (TGF-β) activation under hypoxic conditions [72]. TSP1 is associated with tissue repair and inflammation, and its levels can change in response to inflammatory signals [50]. TSP1 level variations can be relevant in age-related processes and degenerative conditions [73]. Our results show that, due to a lack of B55α in endothelial cells, TSP1 levels (mRNA and protein) were significantly lower. Scratch assays showed impaired wound healing in B55α-depleted cells. It has been reported that the expression of TSP1 is increased in the early phase of wound healing, whereas TSP2 is produced during the proliferative phase of the process [50,74]. However, in our experiment, delayed wound healing was coupled with a lack of TSP1 level increase during wound closure of B55α-depleted cells. 

To provide a physiologically relevant environment for angiogenesis assays, we utilized a Matrigel 3D extracellular matrix and spheroid formation assays. The tube formation assay assesses the ability of endothelial cells to form capillary-like structures, mimicking the early stages of angiogenesis. The extracellular matrix components in Matrigel provide a three-dimensional scaffold that mimics the natural microenvironment, promoting cell adhesion, migration, and the formation of tubular structures resembling blood vessels [75]. The angiogenesis assay demonstrated altered tube-like structures in B55α-depleted cells, emphasizing the role of PP2A B55α in angiogenesis. B55α-depleted cells started to form tube-like structures earlier than control cells with more branching and total segment length. Similarly, it has been reported that the dissociation of the B subunit from the AC dimer of PP2A leads to the inactivation of PP2A and promotes cell migration and angiogenesis [76,77]. Moreover, B55α depletion affected spheroid formation as well. Notably, in the absence of B55α, the disintegration of spheroids was more pronounced compared to those formed by control or nonsiRNA-treated cells, which remained relatively stable. The fragmentation of spheroids refers to the disintegration or breaking apart of the three-dimensional cellular aggregates. It could be an indication that the spheroids formed were not stable enough to maintain their structural integrity. This might be due to factors affecting cell–cell interactions. Maud et al. reported that endothelial cells lacking B55α could initially establish branched networks; however, these networks were unsustainable, as the branches exhibited high instability, ultimately resulting in a swift collapse of the network [77]. 

Subsequently, phosphomimic (S93D) and phosphonull (S93A) TSP1 mutants were created, with spheroid and tube formation assays revealing the impact of the TSP1 phosphorylation state on angiogenic properties. Our findings indicate that TSP1-S93D overexpressing cells formed smaller and more compact spheroids than TSP1 wt or TSP1-S93A expressing cells. Moreover, the TSP1-S93D mutant-containing cells exhibited poor capillary formation. These results suggest that PKC phosphorylation influences the effect of TSP1 angiogenesis. 

The challenges in researching TSP1 stem from its biological complexity, diverse functions, cellular interactions, tissue-specific effects, and clinical relevance. Traditionally, TSP1 has been recognized for its extracellular roles; however, emerging research has suggested that TSP1 may also have intracellular roles, influencing cellular functions inside the cell rather than at the cell surface or in the extracellular matrix [56]. The intracellular functions of TSP1 are still an area of active investigation, and the mechanisms involved are not fully understood. The research is further complicated by the fact that peptides derived from TSP1 and full-length TSP1 can act differently. Peptides can have more a targeted and focused impact on cellular processes; however, full-length TSP1 is more likely to influence a wider range of signaling pathways due to the presence of multiple domains. TSP1’s antiangiogenic effects make it a potential target for anti-cancer therapies, as inhibiting angiogenesis can limit the blood supply to tumors, hindering their growth and metastasis. Full-length TSP1 exhibits limited bioavailability and is prone to proteolytic degradation; its shorter angiostatic peptides are potent and promising therapeutic agents [78]. For example, ABT-510 peptide is derived from the second type 1 repeat of TSP-1. Clinical trials have been conducted to evaluate the safety and efficacy of ABT-510 in various types of cancer, including melanoma and prostate cancer [79,80]. On the other hand, understanding TSP1’s proangiogenic effects in specific contexts is crucial for a comprehensive understanding of its role in the tumor microenvironment. In cardiovascular physiology and pathology, TSP1 can contribute to the development of therapies for conditions such as atherosclerosis, thrombosis, and vascular diseases.

Due to its tumor-suppressive properties, PP2A also has garnered interest as a potential target for cancer therapy. Several small molecule inhibitors have been developed to selectively inhibit PP2A activity in cancer cells. These inhibitors work by binding to specific subunits of the PP2A complex, disrupting its function and promoting tumor cell death. In addition to naturally produced pharmacological inhibitors of PP2A, like okacaid acid [81,82] or cantharidin [83,84], several newly synthesized inhibitors are being tested. LB-100 is a potent small molecule inhibitor of PP2A that has shown promising results in preclinical studies [85]. While PP2A inhibitors hold promise as anticancer agents, their clinical development has been challenging due to the essential role of PP2A in normal cellular function. The challenge lies in achieving selective inhibition of PP2A activity in cancer cells while sparing normal cells.

In summary, the results shown here provide evidence for the intracellular role of PP2A B55α in angiogenesis, specifically through the regulation of TSP1. The findings highlight the intricate interplay between PP2A B55α, PKC, and TSP1 in modulating key cellular processes involved in angiogenesis. The results deliver valuable insights into the molecular mechanisms governing angiogenesis and suggest potential therapeutic targets for angiogenesis-related disorders. While the findings contribute to our understanding of the TSP1 posttranslational modification, function, and potential therapeutic applications, further studies focusing on peptide or phosphopeptide derivatives may offer more targeted and precise insights. By investigating peptide-derived fragments, future research can explore specific functional domains, optimize bioavailability, and enhance therapeutic efficacy.

## 4. Materials and Methods

### 4.1. Reagents

Materials were obtained from the following sources: paraformaldehyde (PFA, P6148), dimethylsulfoxide (DMSO; D2650), PBS tablet (P4417), Tween 20 (P7949), Triton X-100 (T8787), sodium dodecyl sulfate (SDS; 436143), glycerol (G7893), bovine serum albumin (BSA, A9647), phorbol 12-myristate 13-acetate (PMA, P8139), Gö6976 (365250), okadaic acid (OA, O9381), isopropyl β-D-1-thiogalactopyranoside (IPTG, I5502), Brilliant Blue R (27816), and ethylenedinitrilotetraacetic acid (EDTA, E9884) from Sigma (St Louis, MO, USA), tautomycetin (TM; 2305) from Tocris Bioscience (Bristol, UK, Europe), Fast Digest restriction enzymes, T4 DNA ligase (EL0011), and Phusion^®^ High-Fidelity DNA Polymerase (F-530) from Thermo Scientific, Inc. (Vantaa, Finland), tris(hydroxymethyl)aminomethane (TRIS, 33,621.260), sodium chloride (NaCl, 27,788.29), and glycine (10119CU) from VWR International (Radnor, PA, USA), methanol (05730-101-350) and acetic acid (02790-101-350) from Molar Chemicals Kft (Halásztelek, Hungary), and cOmplete^TM^ Mini protease inhibitor cocktail (11836153001) from Roche Diagnostics (Basel, Switzerland). All chemicals were of analytical or molecular biology grade. 

Cell culture reagents as Minimum Essential Medium with Earle’s Salts with L-Glutamine (MEM-A); Fetal Bovine Serum (FBS) collected in South America (FBS-12A) was obtained from Capricorn Scientific GmbH (Ebsdorfergrund, Germany); MEM Non-essential Amino Acid mixture (13-114E), Na-pyruvate (BE13-115E), Trypsin/EDTA solution (CC-5012) was obtained from Lonza Group AG (Basel, Switzerland). 

### 4.2. Cell Culture and Treatments

Bovine pulmonary artery endothelial cells (BPAEC) culture line-CCL 209 (American Type Tissue Culture Collection, Rockville, MD, USA) were maintained as described earlier [86]. Cell cultures were maintained under sterile conditions using a Class II laminar flow hood (Model: NuAire NU 437-400E, NuAire, Plymouth, MN, USA). Cell cultures were incubated at 37 °C in a humidified CO_2_ incubator (Model NU-4850, NuAire, Plymouth, MN, USA). 

PMA, OA, and TM were dissolved in dimethyl-sulfoxide (DMSO) and used in a serum-free medium. Stock concentrations were: 5 mM PMA, 13 mM Gö6976, 1.2 mM OA, and 16 mM TM. With intermediate dilutions using a serum-free medium, the final concentrations used were as follows: 50 nm PMA, 1 μm Gö6976, 5 nm OA, and 1 μm TM. In addition, 0.1% DMSO was used as a control. Chosen concentrations did not exert adverse effects on cell viability as reported by others [87,88,89].

### 4.3. Gene Silencing and Transfection

Gene silencing of PP2A B55α was achieved by using 50 nM of ON-TARGET plus PPP2R2A SMARTpool-siRNA (L-004824, GE-Healthcare Dharmacon Inc., Lafayette, CO, USA) or PP2A-B55-α siRNA (sc-39185, Santa Cruz, Dallas, TX, USA) as described in [48]. BPAEC cells were transfected with pcDNA3.1myc-HisA TSP1 wild type (wt), -TSP1 S93A and -TSP1 S93D plasmids by Lipofectamine 3000 transfection reagent (Invitrogen Corporation, Carlsbad, CA, USA) based on the manufacturer’s protocol (https://www.thermofisher.com/document-connect/document-connect.html?url=https://assets.thermofisher.com/TFS-Assets%2FLSG%2Fmanuals%2Flipofectamine3000_protocol.pdf; accessed on 21 September 2021). 

### 4.4. RT-PCR and Quantitative Real-Time PCR (qPCR) Measurement

Total RNA was isolated using a GeneJET RNA Purification Kit (Thermo Scientific, Waltham, MA, USA). cDNA was synthesized using 500 ng total RNA, oligo-d(T)_16_ primer and RevertAid reverse transcriptase (Thermo Scientific, Waltham, MA, USA). qPCR measurements were performed as described in [90]. The threshold values (Ct values) were normalized to actin or GAPDH. 2^−ΔΔCt^ values were calculated from the normalized data. Primers were synthesized by Integrated DNA Technologies (Coralville, IA, USA): TSP1: 5′-GTCCTGCAGAATGTAAGGTTTG-3′, 5′-GGTGACAAAGACACTGGTAGAG-3′; PP2A B55α: 5′-CAACCGGAGATAAAGGTGGTAG-3′; 5′- GGCTCTGAAAGGTGCTGTAA-3′ GAPDH: 5′-GATGCTGGTGCTGAGTATGT-3′, 5′-GCAGAAGGTGCAGAGATGAT-3′; β-actin: 5′-GCAAGTACTCCGTGTGGATT-3′, 5′-GACTCATCGTACTCCTGCTTG-3′.

### 4.5. SDS-PAGE and Western Blotting

Protein samples were separated by SDS-PAGE and either stained with Coomassie Blue gel staining solution (50% methanol, 10% acetic acid, and 1 g/L Coomassie Brilliant Blue R-250) or transferred to Advantec Cellulose Nitrate 0.45 µm membrane (S045A330R; Life Science Products, Inc., Frederick, CO, USA) as described before in [91]. The antibodies used in this study are listed in Table 1.

### 4.6. Immunoprecipitation, Immunofluorescence Staining, and Proximity Ligation Assay

Immunoprecipitation (IP) and immunofluorescence (IF) staining were carried out as described earlier [86]. For IF staining, primary antibodies were diluted using a ratio of 1:100 in blocking solution, and Alexa 488-, Alexa 594-conjugated secondary antibodies (Molecular Probes, Invitrogen, Carlsbad, CA, USA) were used in 1:300 dilutions. The confocal images were prepared by a Leica TCS SP8 confocal microscope. Proximity ligation assay (PLA) was performed as described in [48].

### 4.7. Anti-V5 Agarose Affinity Gel

Interactions between TSP1 and recombinant V5-tagged PP2A Bα and PP2A B’γ were tested by Anti-V5 Agarose Affinity Gel (A7345; Sigma, St. Louis, MO, USA) as described in [92].

### 4.8. Generation of TSP1 Constructs

The nested sequence of wild-type TSP1 was amplified using Kyratec PCR Super Cycler Instrument (model: SC300G0, Applied Biosystems, Foster City, CA, USA) and the following primer pair: 5′-GGTACACACAAGATCCCTGCTGG-3′, 5′-CCTTAGTGCTTTGGCCTCTGCTCC-3′. Subsequently, the coding sequence of full-length TSP1 was amplified using GoTaq G2 Flexi DNA Polimerase (M7801; Promega, Madison, WI, USA) and specific primers containing SalI (5′-TATGTCGACCATGGGGCTGGCCTGGG-3′) and KpnI (5′-ACTACGGTACCTTAGGGATCTCTACATTCGTATTTC-3′) restriction sites. The amplified sequence was inserted into the pGEM-T Easy vector (A1360; Promega, Madison, WI). After transformation and selection, the coding sequence was cleaved out from the vector using restriction enzymes and inserted into the pGEX-4T-2 vector (Clontech Laboratories Inc. Mountain View, CA, USA). Additional bacterial TSP1 fragment constructs were prepared from the full-length TSP1 construct and inserted into the pGEX-4T-2 vector. Constructs and primers are: pGEX-4T-2-TSP1^1–221^: 5′-TTAGGATCCATGGGGCTGGCCTGGGG-3′, 5′-GTACTCGAGTCAGGTTCCAAAGACAAACCTCAC-3′; pGEX-4T-2-TSP1^222–314^: 5′-TGAATTCCCACACCAGAAGACATCCTCAGG-3′, 5′-GTTCTCGAGCTACCGCCTCAGCTCATTG-3′; pGEX-4T-2-TSP1^315–697^: 5′-TATCCCGGGCCTCCCCTATGCTATCACAA-3′, 5′-TATCTCGAGCTATCATCCAGGTCTGTGTCCTC-3′; pGEX-4T-2-TSP1^698–1171^: 5′-GTTGGATCCGAGGACACAGACCTGGATG-3′, 5′-GACCTCGAGTTAGGGATCTCTACATTCGTATTTC-5′, pGEX-4T-2-TSP1^1−221^ S44A: 5′Phos-CGCAAGGGGGCTGGGCGCCGA-3′, 5′Phos-CGCAAGGGGGATGGGCGCCGA-3′. For pGEX-4T-2-TSP1^1−221^ S44A/S93A double mutant 5′Phos-TTCTGGCAGCCCTGAGGCAGA-3′and 5′Phos-CGTGCCCCGGGTCTTCTTCA-3′ primers were used and pGEX-4T-2-TSP1^1–221^ S44A construct as template.

For mammalian expression of TSP1, the coding sequence was amplified using 5′-TACTCGAGTAATGGGGCTGGCCTGG-3′, 5′-GGCAAGCTTGGGATCTCTACATTCGTATTT-3′ primer pair and cloned into pcDNA3.1 myc-HisA vector (Life Technologies, Carlsbad, CA, USA). Phosphomutant TSP1 constructs were prepared by back-to-back PCR using primers containing site-directed mutagenesis. The primers were pcDNA3.1myc/His A TSP1^S93A^: 5′Phos-TTCTGGCAGCCCTGAGGCAGA-3′, 5′Phos-CGTGCCCCGGGTCTTCTTCA-3′; pcDNA3.1myc/His A TSP1^S93D^: 5′Phos-TTCTGGCAGACCTGAGGCAGA-3′, 5′Phos-CGTGCCCCGGGTCTTCTTCA-3′. All primers were synthesized by Integrated DNA Technologies (Coralville, IA, USA). All constructs were verified by sequencing (BIOMI Kft, Gödöllő, Hungary). 

### 4.9. Bacterial Recombinant Protein Expression and GST Pull down Assay

pGEX-4T-2-TSP1 and pGEX-4T-2-TSP1 fragments were transformed into *Escherichia coli* (*E.coli*) BL21 (DE3) cells. Cells were grown at 37 °C and shaken at 180 rpm using Forma Scientific Incubated Orbital Shaker (model 4535; Forma Scientific, Marietta, OH, USA), until O.D._600_ = 0.5, measured with Genesys 20 spectrophotometer (Z376027; Merck, Darmstadt, Germany). Then, different concentrations of IPTG were added to induce protein production. After the optimization of each protein used final IPTG concentration, the temperature and time for induction were as follows: pGEX-4T-2-TSP1^1–221^, pGEX-4T-2-TSP1^1–221^ S44A, pGEX-4T-2-TSP1^1–221^ S93A, pGEX-4T-2-TSP1^1–221^ S44A/S93A: 0.1 mM, 13 °C, O/N, pGEX-4T-2-TSP1^222–314^: 1 mM, 37 °C, 3 h, pGEX-4T-2-TSP1^315–697^: 0.1 mM, RT, O/N, pGEX-4T-2-TSP1^698–1171^: 0.5 mM, RT, 3 h. The GST-pull down assay was described earlier in [91].

### 4.10. In Vitro PKC Kinase Assay

Purified GST-TSP1 fragments immobilized on Protino Glutathione Agarose 4B beads (745500.10, Macherey-Nagel, Düren, Germany) were incubated with Kinase Assay Buffer (25 mM MOPS, 12.5 mM β-glycerol-phosphate, 25 mM MgCl_2_, 5 mM EGTA, 2 mM EDTA, 0.25 mM DTT) with or without active PKCα (P61-18G; SignalChem, Richmond, BC, Canada) for 2 h at 30 °C in a water bath (model: WB7, MEMMERT GmbH, Schwabach, Germany). Samples were washed with TBS (25 mM Tris-HCl, 0.15 M NaCl, pH 7.5) and either boiled with 2× sample buffer (4% SDS, 20% glycerol, 120 mM Tris-HCl, pH 6.8) and tested with SDS-PAGE or Western blot or further used in dephosphorylation assay. 

### 4.11. Proteome Profiler™ Human Angiogenesis Antibody Array

Proteome Profiler Human Angiogenesis Antibody Array (ARY007) was ordered from R&D Systems, Inc. (Minneapolis, MN, USA). The protein concentration of nonsiRNA and siPPP2R2A-treated cell lysate was measured and an equal amount was used according to the manufacturer’s description (https://resources.rndsystems.com/pdfs/datasheets/ary007.pdf?v=20240127&_ga=2.71900629.713239171.1706435623-84994310.1701775422, accessed on 17 January 2021). Membranes were imaged for 30 s simultaneously with the ChemiDoc^TM^ Touch Imaging System (Bio-Rad, Hercules, CA, USA). Pixel densities were evaluated by the ImageJ software (version 1.54h). 

### 4.12. Matrigel In Vitro Tube Formation Assay

To study the effect of the gene silencing or TSP1 overexpression on the capillary tube formation of the endothelial cells, 1 × 10^3^ cells were seeded onto μ-Slide angiogenesis, ibiTreat plates (Ibidi, Gräfelfing, Germany) previously coated with BD Matrigel Basement Matrix (354234, BD Biosciences, Franklin Lakes, NJ, USA). Images were taken at different timepoints by a Leica MC 120 HD microscope. The tube formation was evaluated by the ImageJ software (version 1.54h), Angiogenesis Analyzer Tool (accessed on 15 January 2023).

### 4.13. Scratch Assay

The confluent monolayer of cells was scratched with 1000 µL filter-tip (70.3050.255, Sarstedt, Nürnbrecht, Germany) in PBS (20 mM Na_2_HPO_4_, 115 mM NaCl, pH 7.4). After scratching, PBS was removed, and fresh complete media was added. Images were taken at different timepoints with a Leica microscope (model: DM IL LED, Leica, Wetzlar, Germany) equipped with a Leica MC120 HD camera (Leica, Wetzlar, Germany). Wound healing closure was evaluated using the ImageJ software.

### 4.14. Magnetic 3D Cell Culturing

For the 3D cell culturing 96-well Bioprinting Kit (Greiner Bio-One, Kremsmünster, Austria) was used. Magnetization and spheroid formation were performed according to the manufacturer’s protocol (https://www.gbo.com/fileadmin/imported_from_old/U072108_IFU_655840_96-Well_Bioprinting_Kit_clear_Rev02.pdf, accessed on 5 May 2022). Briefly, NanoShuttle was added to the cells (1 μL/10,000 cells) and incubated overnight in a humidified CO_2_ incubator (Model NU-4850, NuAire, Plymouth, MN, USA) at 37 °C. After incubation, the magnetized cells were seeded into a cell-repellent 96-well plate (655970; Greiner Bio-One, Kremsmünster, Austria). Pictures were taken at different timepoints after the removal of the magnetic plate with a Leica microscope (model: DM IL LED, Leica, Wetzlar, Germany) equipped with a Leica MC120 HD camera (Leica, Wetzlar, Germany). The size of the spheroids was evaluated using the ImageJ software.

### 4.15. Statistical Analysis

Results are presented as means ± SD. Statistical analysis was performed with GraphPad Prism (version 8.0.1) by the Dotmatics (GraphPad Software, Boston, MA, USA) program using different tests specified for each experiment (see figure legends), with differences considered significant for *p* < 0.05 (*), *p* < 0.01 (**), and *p* < 0.001 (***). Densitometry of immunoblots was performed by the ImageJ software (version 1.54h) [93]. 

## Figures and Tables

**Table 1:** Antibodies utilized in Western blot.

Antibody	Dilution	Vendor (cat#)
PP2A Bα subunit (100C1)	1:1000	Cell Signaling Technologies (Danvers, MA, USA) (#2290)
PPP2R2A (2G9)	1:1000	Cell Signaling Technologies (Danvers, MA, USA) (#5689)
PP2A A subunit	1:1000	Cell Signaling Technologies (Danvers, MA, USA) (#2039)
PP2A C subunit	1:1000	Cell Signaling Technologies (Danvers, MA, USA) (#2038)
phospho-(Ser) PKC substrate	1:1000	Cell Signaling Technologies (Danvers, MA, USA) (#2261)
thrombospondin-1	1:1000	Cell Signaling Technologies (Danvers, MA, USA)(#14778)
thrombospondin-1 (C-8)	1:1000	Santa Cruz Biotechnology (Dallas, TX, USA) (sc-393504)
actin	1:1000	Sigma (St. Louis, MO, USA) (A5060)
V5-tag (D3H8Q)	1:1000	Cell Signaling Technologies (Danvers, MA, USA) (#13202)
V5-tag	1:1000	Thermo Scientific (Vantaa, Finland) (R960-25)
c-myc	1:1000	Invitrogen (Carlsbad, CA, USA) (13-2500)
anti-rabbit IgG HRP-linked	1:5000	Cell Signaling Technologies (Danvers, MA, USA) (#7074)
anti-mouse IgG HRP-linked	1:5000	Cell Signaling Technologies (Danvers, MA, USA) (#7076)

## Data Availability

The data that support the findings of this study are available from the corresponding author upon reasonable request.

## Supplementary Materials

The following supporting information can be downloaded at: https://www.mdpi.com/article/10.3390/ijms25031844/s1.
