# Supplementary material for: PP2A Affects Angiogenesis via Its Interaction with a Novel Phosphorylation Site of TSP1
Source: Int J Mol Sci. 2024 Feb 3;25(3):1844. doi: 10.3390/ijms25031844 (PMC10855381; doi:10.3390/ijms25031844)
Supplement: Supplementary file 1 [file ijms-25-01844-s001.zip › Supplementary files.pdf]

Supplementary files:

Suppl. Figure 1.

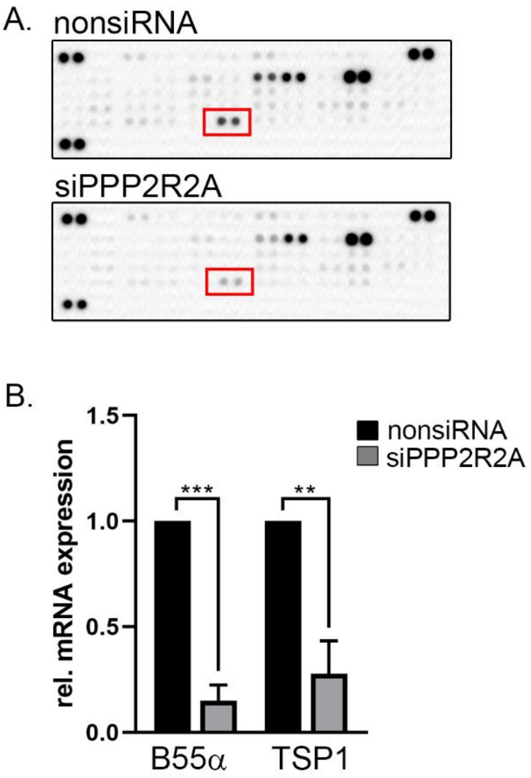

Supplementary Figure S1. Depletion of B55*α* in endothelial cells (A) Cell lysates of non-targeting siRNA and B55*α* specific siRNA (siPPP2R2A) treated BPAEC were incubated on angiogenetic specific antibodies containing proteome profiler array membrane. Membranes were imaged for 30 secs simultaneously. Red rectangles mark dots corresponding of TSP1 signals. (B) qPCR measurements were made using mRNA from nonsiRNA and PP2A-B55-*α* siRNA (sc-39185, Santa Cruz) (siPPP2R2A) BPAEC cells. Quantitative analysis of B55*α* and TSP1 signals are shown. Actin were used for mRNA level normalization. Significant changes were determined by unpaired t-test (n=3; *** $p < 0.001$).

Suppl. Figure 2.

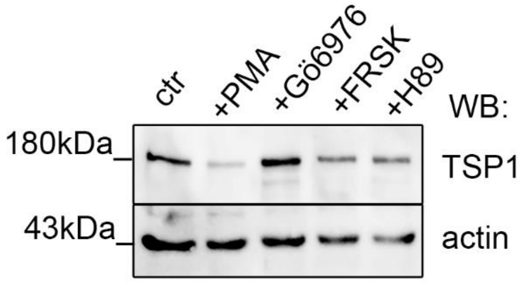

Supplementary Figure S2. Effect of PKA and PKC activity on TSP1 protein level BPAEC cells were treated with phorbol myristate acetate (PMA), Gö6976 (a PKC inhibitor), forskolin (FRSK) or H89 (PKA inhibitor) for 12h and tested in Western blot for TSP1 and actin.

Suppl. Figure 3.

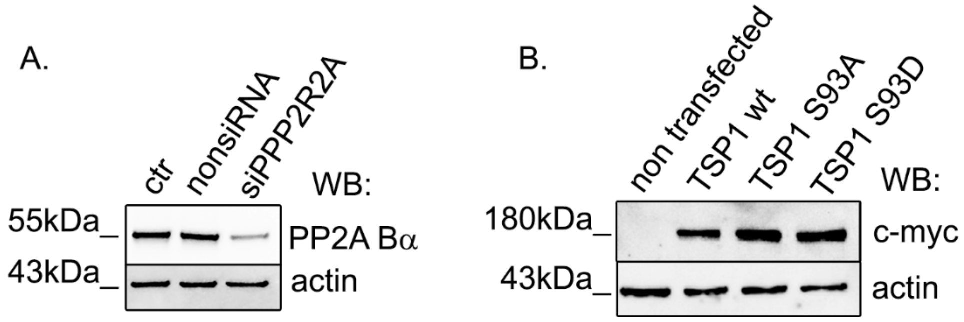

Supplementary Figure S3. Western blot analysis of 3D spheroids (A) NanoShuttle magnetic beads were added to the control, nonsiRNA and siPPP2R2A treated cells. Efficiency of silencing was confirmed by Western blot using the magnetized cells. (B) BPAEC cells were transfected with pcDNA3.1 myc-HisA TSP1 wt, -S93A or -S93D plasmids. Overexpression of TSP1 was detected using c-myc specific antibody. Actin was used as loading control
