# Supplementary figures and images for: PP2A Affects Angiogenesis via Its Interaction with a Novel Phosphorylation Site of TSP1
Source: Int J Mol Sci. 2024 Feb 3;25(3):1844. doi: 10.3390/ijms25031844 (PMC10855381; doi:10.3390/ijms25031844)

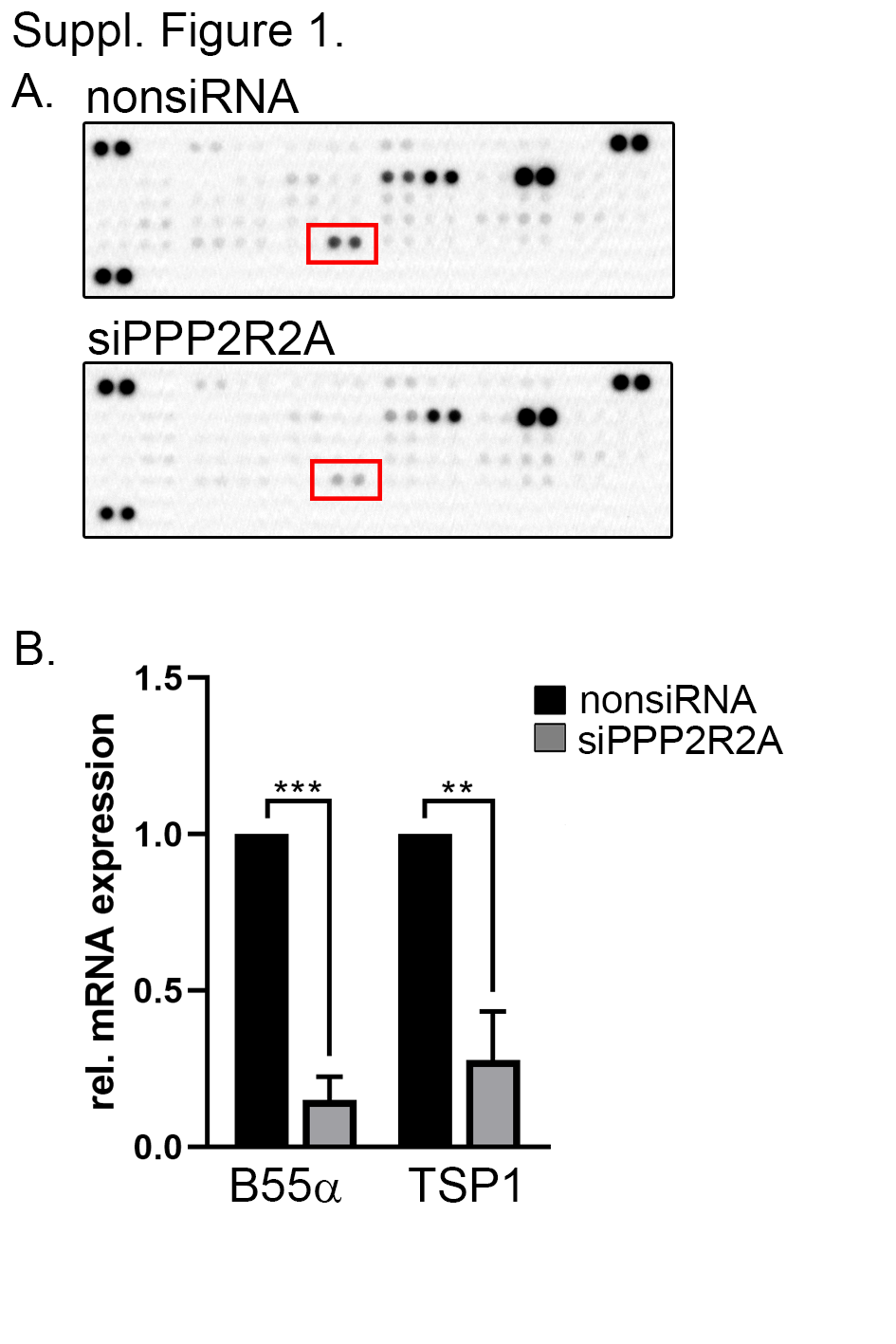

Supplement: Supplementary file 1 [file ijms-25-01844-s001.zip › supplementary figure 1.tif]

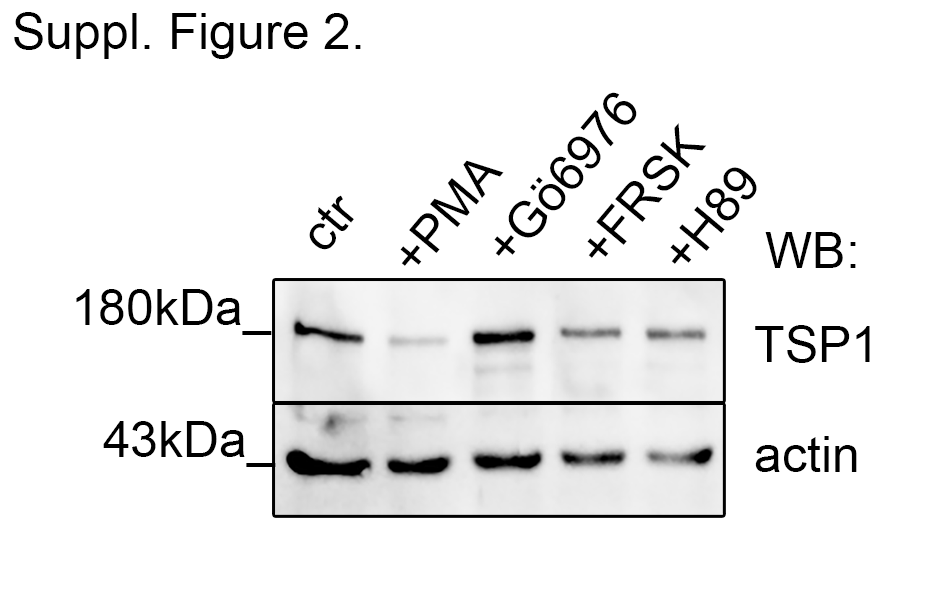

Supplement: Supplementary file 1 [file ijms-25-01844-s001.zip › supplementary figure 2.tif]

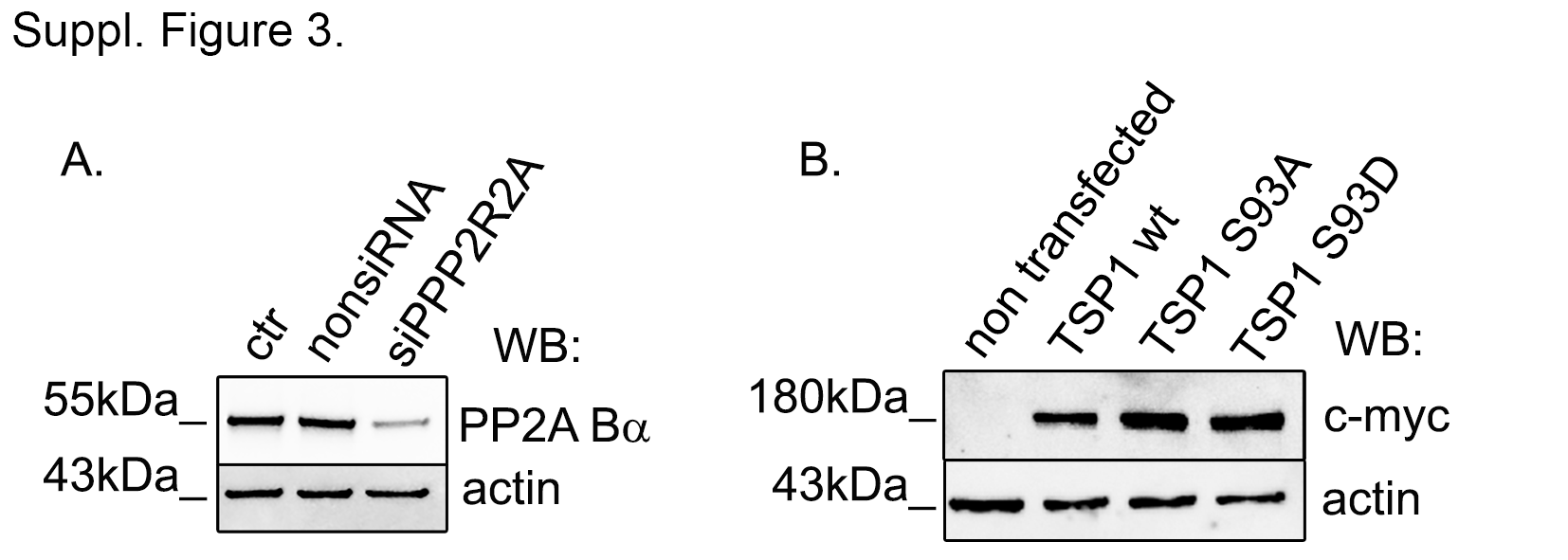

Supplement: Supplementary file 1 [file ijms-25-01844-s001.zip › supplementary figure 3.tif]
